# In-Cell Testing of Zinc-Dependent Histone Deacetylase Inhibitors in the Presence of Class-Selective Fluorogenic Substrates: Potential and Limitations of the Method

**DOI:** 10.3390/biomedicines12061203

**Published:** 2024-05-29

**Authors:** Alla Kleymenova, Anastasia Zemskaya, Sergey Kochetkov, Maxim Kozlov

**Affiliations:** Engelhardt Institute of Molecular Biology, Russian Academy of Sciences, 119991 Moscow, Russia; kaamail@rambler.ru (A.K.); a.zemskaia@mail.ru (A.Z.); snk1952@gmail.com (S.K.)

**Keywords:** HDAC inhibitors, fluorogenic substrates, class selectivity, correctness/incorrectness of testing results

## Abstract

The development of anticancer drugs based on zinc-dependent histone deacetylase inhibitors (HDACi) has acquired great practical significance over the past decade. The most important HDACi characteristics are selectivity and strength of inhibition since they determine the mechanisms of therapeutic action. For in-cell testing of the selectivity of de novo-synthesized HDACi, Western blot analysis of the level of acetylation of bona fide protein substrates of HDACs of each class is usually used. However, the high labor intensity of this method prevents its widespread use in inhibitor screening. We developed an in-cell high-throughput screening method based on the use of three subtype-selective fluorogenic substrates of the general structure Boc-Lys(Acyl)-AMC, which in many cases makes it possible to determine the selectivity of HDACi at the class level. However, we found that the additional inhibitory activity of HDACi against metallo-β-lactamase domain-containing protein 2 (MBLAC2) leads to testing errors.

## 1. Introduction

The superfamily of histone deacetylases, including Zn^2+^-dependent HDACs and NAD^+^-dependent SIRTs, catalyzes the removal of acyl groups from lysine residues of a variety of substrate proteins, while histone acetyltransferases (HATs) carry out the opposite transformation. Moreover, in cells, especially in mitochondria, there is non-enzymatic N-acylation of proteins with acetyl- or succinyl-coenzyme A, both free [1] and in complex with the GCN5L1 protein, which does not have target-selective acyltransferase activity [2].

Based on substrate selectivity and functional characteristics, HDACs are divided into three classes: I, II (a/b), and IV. HDAC1/2/3/8 belonging to class I are mainly localized in the nucleus and play a key role in the regulation of gene expression by deacylating histones and transcription factors. Out of the four isoenzymes of this class, HDAC1/2 function as part of the corepressor complexes NuRD, CoREST, MiDAC, and SIN3, while HDAC3 functions as part of the SMRT/NCoR complex [3,4]. Class IIa is represented by regulatory isoforms of HDAC4/5/7/9, which, despite weak catalytic activity, are involved in transcriptional repression by interacting with HDAC3 or the transcription factor MEF2 [5,6]. Class IIb includes HDAC6/10, which are mainly localized in the cytosol. The quantitatively predominant HDAC6 that has affinity for numerous intermediary proteins deacetylates cytoskeletal, heat shock, and many other proteins [7,8,9]. The main substrates of HDAC10 are acetylated polyamines [10]. Finally, HDAC class IV is represented by a single isoform, HDAC11: the only fatty acid deacylase, the activity of which in relation to acyl residues C12–C18 is four orders of magnitude higher than the acetyl group [11,12].

Many HDAC inhibitors (HDACi) affect the proliferative and metabolic status of target cells through pleiotropic epigenetic effects, which often have high therapeutic potential [13]. The most important characteristics of HDACi are selectivity and strength of inhibition since they determine the mechanisms of therapeutic action [14]. Currently, there are five HDACi that have been approved for clinical use as anticancer drugs: Vorinostat, Romidepsin, Panobinostat, Belinostat, and Tucidinostat [15]. Among these compounds, only Panobinostat and Belinostat are universal inhibitors of HDACs of all classes (pan-inhibitors); all other compounds are class-selective to varying degrees.

For in vitro testing of HDACi selectivity and potency, recombinant HDACs and their fluorogenic substrates, namely lysine derivatives or various peptides (some of which were described for the first time two decades ago), are most often used [16,17]. Currently, the most popular and commercially available fluorogenic HDAC substrates have become Boc-Lys(Ac)-AMC (substrate selectivity for HDAC1/2/3 class I and HDAC6 class IIb) and Boc-Lys(Tfa)-AMC (substrate selectivity for HDAC class IIa and to a lesser extent for HDAC8/10/11) [18,19]. In both cases, enzymatic removal of the acyl group from the substrate results in the formation of a product, Boc-Lys-AMC (Prod^Lys^), which, after incubation with trypsin, is detected by the release of brightly fluorescent 7-amino-4-methylcoumarin (AMC) [17].

A two-substrate method to determine the class selectivity of HDACi (class I/IIb or class IIa) was implemented in complex mixtures of HDACs derived from nuclear or cell lysates. Unlike individual recombinant HDACs, cell lysates contain all classes of HDACs in the form of multicomponent complexes characteristic of living cells [20,21]. There is no doubt that the ideal source of HDAC activity for HDACi testing is a living cell, and this circumstance has largely stimulated the development of appropriate in-cell techniques [22,23,24,25,26,27]. However, a comparative in-cell testing of HDACi using only two HDAC substrates, i.e., Boc-Lys(Ac)-AMC (Sub^Ac^) and Boc-Lys(Tfa)-AMC (Sub^Tfa^), does not allow distinguishing the selectivity of inhibitors against HDAC1/2/3 class I and HDAC6 class IIb. In this work, we suggest a three-substrate version of the cell-test system (s^3^CTS) for correct assessment of the strength and class selectivity of HDACi and also analyzed the limitations of the method imposed by the structure of the inhibitors.

## 2. Materials and Methods

### 2.1. Cell Culture

HCT116 cells were grown in the DMEM medium supplemented with 10% FBS (BioSera, Cholet, France), 2 mM L-glutamine, 100 U/mL penicillin, and 100 μg/mL streptomycin in the presence of 5% CO_2_ at 37 °C. Cells were re-seeded every three days at a ratio of 1:3 or 1:5.

### 2.2. Inhibitors, Substrates and Antibodies

HDAC inhibitors, namely Belinostat (S1085), Bufexamac (S3023), Nexturastat A (S7473), Panobinostat (S1030), Tacedinaline/CI-994 (S2818), TMP-269 (S7324), Tubastatin A (S8049), UF010 (S5810), Vorinostat (S1047), and WT161 (S8495), were all from Selleckchem (Houston, TX, USA); Cmpd13 was synthesized as described in [28]. Substrates, namely Boc-Lys(Ac)-AMC (4033972), Boc-Lys(Tfa)-AMC (4060676), and Boc-Lys-AMC (4033973), were all from Bachem (Switzerland). The stock solutions of test compounds (100 mM in DMSO unless stated otherwise) were serially diluted with DMSO to 100× concentrations; the corresponding 100× solution was added to each well of a culture plate in an amount of 1% of its volume. Vehicle control samples were treated with the same amount of DMSO. The following antibodies were used: rabbit anti-histone H3 (9715S) and rabbit anti-acetylated K9/14 histone H3 (9677S) were from CST (Danvers, MA, USA); rabbit anti-α-tubulin K40 acetylated (sab5600134) was from Abcam (Waltham, MA, USA); mouse anti-α-tubulin (T5168) was from Sigma (Livonia, MI, USA). Conjugates of horseradish peroxidase with secondary specific antibodies (anti-mouse (sc-2005) and anti-rabbit (sc-2004)) were from SCBT (Dallas, TX, USA).

### 2.3. Synthesis of Fluorogenic Substrates Boc-Lys(Cro/Pro)-AMC

(*i*) DBU (0.61 g, 4 mmol) was added to a suspension of Boc-Lys-OH (0.98 g, 4 mmol) in MeOH (8 mL). After almost complete dissolution of the slurry, the corresponding Cro/Pro-NHS-ether (4–5 mmol) was added with intensive mixing. The reaction mixture was left for 3 h at room temperature; then, the solvent was evaporated, the residue was mixed with 0.4 M HCl (15 mL) and washed twice with 20 mL and 15 mL EtOAc, and the organic layers were combined and evaporated. The products were isolated by silica gel chromatography using a mixture of CHCl_3_-EtOH-AcOH (100:10:1), and after eluent evaporation, compounds were dried in vacuum to yield Boc-Lys(Cro)-OH (45%) or Boc-Lys(Pro)-AMC (54%).

*Boc-Lys(Cro)-OH*: ^1^H NMR (300 MHz, DMSO-*d*_6_) δ 12.35 (s, 1H), 7.83 (t, *J* = 5.2 Hz, 1H), 7.00 (d, *J* = 7.9 Hz, 1H), 6.59 (dq, *J* = 15.7, 6.8 Hz, 1H), 5.88 (dd, *J* = 15.3, 1.6 Hz, 1H), 3.83 (dt, *J* = 8.7, 4.2 Hz, 1H), 3.08 (dt, *J* = 6.2, 5.9 Hz, 2H), 1.78 (dd, *J* = 6.8, 1.3 Hz, 3H), 1.67–1.51 (m, 2H), 1.46–1.23 (m, 13H). ^13^C NMR (75 MHz, DMSO-*d*_6_) δ 174.68, 165.21, 156.06, 137.72, 126.49, 78.39, 53.89, 38.59, 30.90, 29.26, 28.67, 23.56, 17.73.

*Boc-Lys(Pro)-OH*: ^1^H NMR (300 MHz, DMSO-*d*_6_) δ 12.34 (s, 1H), 7.68 (s, 1H), 6.98 (d, *J* = 7.7 Hz, 1H), 3.83 (dt, *J* = 7.6, 4.3 Hz, 1H), 3.01 (d, *J* = 5.7 Hz, 2H), 2.05 (q, *J* = 7.5 Hz, 2H), 1.75–1.20 (m, 15H), 0.99 (t, *J* = 7.6 Hz, 3H). ^13^C NMR (75 MHz, DMSO-*d*_6_) δ 174.67, 173.11, 156.06, 78.39, 53.88, 38.62, 30.92, 29.23, 28.98, 28.68, 23.52, 10.45.

*(ii)* DCC (113 mg, 0.55 mmol) was added to a chilled and stirred solution of Boc-Lys(Cro/Pro)-OH (1 mmol) in DCM or the mixture of DCM/MeCN (4–8 mL total volume). After 1 h in an ice bath, AMC (88 mg, 0.5 mmol) was added to the mixture. The suspension was stirred at room temperature overnight; then, the dicyclohexylurea was filtered and washed with MeCN. After concentrating the filtrate, the products were isolated by silica gel chromatography using a mixture of CHCl_3_-MeCN-EtOH (35:5:1) in the case of crotonyl derivative and a mixture of CHCl_3_-MeCN (1:2) in the case of propionyl derivative, and after eluent evaporation, compounds were dried in vacuum to yield Boc-Lys(Cro)-AMC (52%) and Boc-Lys(Pro)-AMC (33%).

*Boc-Lys(Cro)-AMC (Sub^Cro^)*: ^1^H NMR (300 MHz, DMSO-*d*_6_) δ 10.39 (s, 1H), 7.83 (t, *J* = 5.2 Hz, 1H), 7.77 (d, *J* = 1.7 Hz, 1H), 7.73 (d, *J* = 8.7 Hz, 1H), 7.50 (d, *J* = 8.5 Hz, 1H), 7.09 (d, *J* = 7.5 Hz, 1H), 6.57 (dq, *J* = 15.6, 6.9 Hz, 1H), 6.27 (s, 1H), 5.86 (dd, *J* = 15.3, 1.4 Hz, 1H), 4.05 (dt, *J* = 8.1, 5.7 Hz, 1H), 3.09 (dt, *J* = 7.0, 5.2 Hz, 2H), 2.41 (s, 3H), 1.76 (dd, *J* = 6.7, 1.0 Hz, 3H), 1.70–1.57 (m, 2H), 1.50–1.21 (m, 13H). ^13^C NMR (75 MHz, DMSO-*d*_6_) δ 172.73, 165.23, 160.48, 156.04, 154.13, 153.56, 142.80, 137.73, 126.47, 115.70, 115.47, 112.73, 106.13, 78.60, 55.75, 38.58, 31.73, 29.39, 28.67, 23.58, 18.43, 17.72.

*Boc-Lys(Pro)-AMC (Sub^Pro^)*: ^1^H NMR (300 MHz, DMSO-*d*_6_) δ 10.38 (s, 1H), 7.88–7.63 (m, 3H), 7.50 (d, *J* = 7.8 Hz, 1H), 7.07 (d, *J* = 5.9 Hz, 1H), 6.26 (s, 1H), 4.06 (s, 1H), 3.02 (d, *J* = 4.5 Hz, 2H), 2.40 (s, 3H), 2.04 (q, *J* = 7.5 Hz, 2H), 1.63 (s, 2H), 1.39 (s, 13H), 0.97 (t, *J* = 7.4 Hz, 3H). ^13^C NMR (75 MHz, DMSO-*d*_6_) δ 173.12, 172.74, 160.47, 156.02, 154.14, 153.53, 142.80, 126.38, 115.70, 115.47, 112.73, 106.13, 78.61, 55.74, 38.62, 31.75, 29.37, 28.98, 28.66, 23.52, 18.42, 10.43.

### 2.4. HDAC Selectivity Assay

HCT116 cells were seeded into 96-well culture plates at a density of 1.5 × 10^4^ cells per well; 24 h after seeding (90–100% monolayer), cells were incubated with various concentrations of the test inhibitors for 24 h; then, three-quarters of the volume from each well was removed and replaced with the same volume containing both the inhibitor at the same concentration and one of three substrates, i.e., Sub^Ac/Pro/Tfa^, at a concentration of 30 µM. After an additional 4 h incubation, aliquots of the culture medium were transferred into a black plate suitable for fluorescence measurement (SPL Life Sciences, Pocheon, Republic of Korea), double-diluted with a solution of 2 mg/mL trypsin in Tris-HCl buffer at pH = 8, and incubated for 60 min at 37 °C. The fluorescence was measured using a Spark multifunctional plate reader (Tecan Trading, Männedorf, Switzerland) at ex/em wavelengths of 360/470 nm.

The fluorescence intensity in each well was then normalized to the cytotoxicity data obtained for the same well. The average normalized fluorescence for each concentration of each test compound was calculated according to the formula below.

$$\mathrm{RFU}=\frac{\sum_{n}\left(\frac{F_{i}-F_{0}}{C_{v}}\right)}{n};$$

where F_i_ is the fluorescence value in a test well, F_0_ is the fluorescence value in a well with medium and without cells, C_v_ is the cell viability, and n is the number of replicates.

### 2.5. Cell Viability Assay

After aliquot transfer to a well, cell viability was determined using the Cell Proliferation Kit I (MTT assay) as specified by the manufacturer (Sigma-Aldrich, St. Louis, MO, USA).

### 2.6. Western Blotting

Western blot analysis was performed essentially according to a previously published procedure [28]. Briefly, cells were seeded into 6-well culture plates at a density of 1.4 × 10^5^ cells per well, cultured for 24 h (40–50% monolayer), and treated with the indicated concentrations of compounds for the specified time. After that, the medium was removed, and the cells were washed with PBS and lysed with a lysis reagent (Promega, Madison, WI, USA). Proteins from the supernatants were separated by electrophoresis in PAAG of a suitable percentage and electrotransferred onto a nitrocellulose membrane. The membrane was treated with 5% dry milk (Bio-Rad, Hercules, CA, USA) in PBST for 60 min at room temperature. Primary antibodies to acetylated K9/14 histone H3 (1:2000), histone H3 (1:4000), K40 acetylated α-tubulin (1:3000), and α-tubulin (1:7000) were added to the membrane, followed by incubation overnight at 4 °C and washing with PBST. A corresponding conjugate of horseradish peroxidase with secondary specific antibodies (1:8000) was added to the membrane, followed by incubation for 60 min at room temperature. The membrane was then washed with PBST, and the signal was visualized using the ECL kit (Pierce Thermo Scientific, Waltham, MA, USA) and a ChemiDoc imaging system (Bio-Rad, Hercules, CA, USA).

### 2.7. Calculation of Correlation and Reliability

Since the original Rank1 and Rank2 series contain the same values, the calculation of the Spearman rank correlation coefficient was carried out according to the formula that included correction for repetitions (in this case, identical variants were assigned an average rank) [29]:

$$r_{S}=\frac{\frac{\left(n^{3}-n\right)}{6}-(T_{x}+T_{y})-\sum d^{2}}{\sqrt{\left(\frac{\left(n^{3}-n\right)}{6}-2\cdot T_{x}\right)\left(\frac{\left(n^{3}-n\right)}{6}-2\cdot T_{y}\right)}}$$

where *n* is the sample size; *d* is the difference between the ranks of conjugate values of features *x* and *y*; *T_x_* and *T_y_* are the corrections for a series of replicates for each sample:

$$T_{x}=\frac{\sum^{kx}\left({t_{x}}^{3}-t_{x}\right)}{12}T_{y}=\frac{\sum^{ky}\left({t_{y}}^{3}-t_{y}\right)}{12}$$

where *t* is the number of members in each group of identical variants. Corrections *T_x_* and *T_y_* take into account *kx* and *ky* groups of repeating variants.

The statistical error and the criterion of reliability of the difference between the correlation coefficient and zero were calculated using the following formulas:

$$m_{r}=\sqrt{\frac{1-r_{S}^{2}}{n-2}}\;\mathrm{and}\;t_{r}=r_{S}m_{r}$$

### 2.8. Statistical Data Analysis

All data are presented as the mean and standard deviation of at least three independent replicates. Statistical significance (assessed by ANOVA with Dunnett’s post hoc test) is shown in the figures: **** (*p* < 0.001), *** (0.001 < *p* < 0.01), ** (0.01 < *p* < 0.05), * (0.05 < *p* < 0.1), and ns—not significant.

## 3. Results and Discussion

### 3.1. Selection and Synthesis of Fluorogenic Substrates of HDACs

The acyl group in lysine derivatives, i.e., artificial HDAC substrates with the general structure Boc-Lys(Acyl)-AMC (Sub^Acyl^), plays a key role in substrate recognition by HDACs of different classes [30]. As noted above, testing of HDACi selectivity can be carried out in living cells used as a “natural reservoir” of HDACs since cell membranes are transparent to Sub^Ac/Tfa^ substrates and the product of their deacylation Prod^Lys^ [23]. An important argument in favor of Sub^Ac/Tfa^ as selective HDAC substrates is the observation that histone deacetylases of the SIRT family deacetylate Ac-N^ε^-Lys-containing peptides two orders of magnitude slower than HDACs [31], whereas Tfa-N^ε^-Lys residues in modified peptides are not deacylated at all [32].

**Scheme 1:**
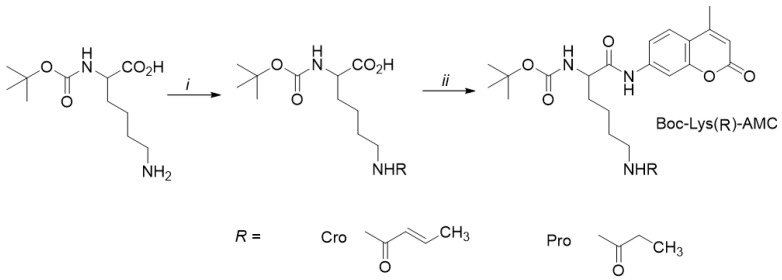
Reagents and conditions: (*i*) Cro-NHS/Pro-NHS, DBU, MeOH, 2–4 h, r.t., yields 45% and 54%, respectively; (*ii*) DCC, CH_2_Cl_2_/MeCN, 0 °C, 1 h, followed by AMC overnight at r.t., yields 55% and 33%, respectively.

Crotonyl and propionyl derivatives Boc-Lys(Cro)-AMC (Sub^Cro^) and Boc-Lys(Pro)-AMC (Sub^Pro^) were previously described as selective substrates of recombinant HDACs class I [30], but both compounds are not commercially available. The use of Sub^Cro/Pro^, Sub^Tfa^, and Sub^Ac^ substrates, which are selective for HDACs class I, class IIA, and classes I/IIB, would theoretically allow identification of the class selectivity of any HDACi when tested in living cells. To implement this approach, we carried out the synthesis of Sub^Cro/Pro^, starting from Boc-Lys-OH (Scheme 1). At the first stage, N^ε^-acylation of Boc-Lys-OH was carried out in MeOH in the presence of the corresponding NHS esters of crotonic and propionic acids and DBU as a base. At the second stage, we carried out the condensation of Boc-Lys(Cro/Pro)-OH with AMC using DCC, as described in the work [33]. Target HDAC substrates Sub^Cro/Pro^ were purified by chromatography on silica gel and crystallization from a suitable solvent. Out of the two substrates, Sub^Pro^, was chosen after testing for further work since incubation of cells in the presence of Sub^Cro^, all other conditions being equal, produced a level of the Prod^Lys^ fluorescent signal several times lower.

### 3.2. Development of Three-Substrate Cell-Test System (s^3^CTS)

#### 3.2.1. Optimization of Conditions for Determining HDAC in Cell Activity

The eukaryotic cell can be considered as a natural “HDAC reactor”, in which the influx of synthetic substrates based on lysine Sub^Acyl^ and the outflow of their N^ε^-deacylation product Prod^Lys^ is guaranteed by the transparency of the membrane [23]. When choosing the optimal conditions for HDACi testing, we relied on data from several works [22,23,27]; however, we excluded the cell lysis stage from the protocol and determined HDAC activity by the content of Prod^Lys^ only in the culture medium; the appropriateness of this approach was proven in further experiments.

**Figure 1:**
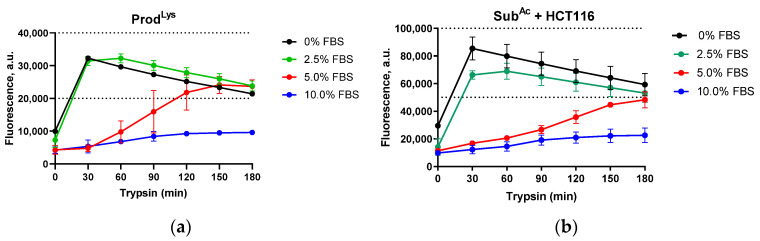
Effect of FBS (0, 2.5, 5, and 10%) on the generation of AMC fluorescent signal in a mixture of trypsin and Prod^Lys^. (**a**) Increase in fluorescence upon digestion of Prod^Lys^ (10 μM) with trypsin (1 mg/mL) in cell medium (0–10% FBS); (**b**) increase in fluorescence upon treatment of culture media with trypsin (1 mg/mL) after a 24 h incubation of HCT116 cells in the presence of Sub^Ac^ (30 μM).

In addition, in the testing protocol at the stage of transition from incubation with inhibitors to incubation with substrates and then with trypsin, we introduced the replacement of the cell medium containing 10% FBS with a medium containing 2.5% FBS. The reason for this was that the suppression of the fluorescent signal by serum proteins described earlier [22] was shown to result primarily from the inhibition of trypsin activity but not from the suppression of cellular HDAC activity. By treating a solution of synthetic Prod^Lys^ in the DMEM medium containing different concentrations of FBS with trypsin, we found that it is the percentage of FBS that determines the kinetics of AMC accumulation (Figure 1a). It is significant that it almost coincides with the kinetics observed when culture media are treated with trypsin after a 24 h incubation of HCT116 cells with Sub^Ac^ (Figure 1b). The drop in fluorescence signal observed in Figure 1a,b at FBS concentrations of 0% and 2.5% can easily be explained by the burnout of AMC as a result of repeated measurements. Thus, decreasing the percentage of FBS allowed us to dramatically increase trypsin activity at the final stage of the test and thereby dramatically reduce the Sub^Acyl^ concentration compared to earlier protocols without greatly compromising the accuracy of the method [22,23,27].

#### 3.2.2. Validation of s^3^CTS by Testing HDACi of Known Selectivity

**Figure 2:**
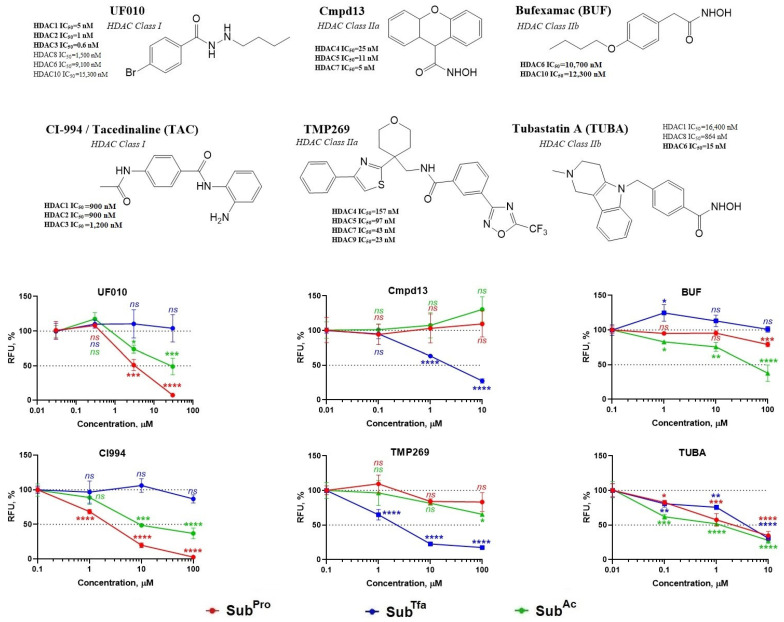
Structures with published IC_50_ data and results of testing class-selective HDACi with s^3^CTS. Statistical significance assessed by ANOVA with Dunnett’s post hoc test: **** (*p* < 0.001), *** (0.001 < *p* < 0.01), ** (0.01 < *p* < 0.05), * (0.05 < *p* < 0.1), and ns—not significant.

To our knowledge, the substrate selectivity of Sub^Pro^ for HDACs class I in cellular systems has not been previously studied. Using HCT116 colon cancer cells that exhibited high HDAC activity under test conditions, we showed that Sub^Pro^ deacylation was almost completely inhibited in the presence of UF010 [34] and CI-994/Tacedinaline (TAC) [35], two selective inhibitors of HDAC1/2/3 (Figure 2). Selective inhibitors of HDAC class IIa, namely Cmpd13 [36], and TMP269 [25], as well as a selective inhibitor of HDAC6/10 class IIb, i.e., Bufexamac (BUF) [37], did not affect the deacylation of Sub^Pro^.

As should be expected, UF010 and TAC even at high concentrations did not completely reduce the level of Sub^Ac^ deacetylation, but did so only by approximately half since the remaining contribution to substrate deacetylation apparently came from the activity of HDAC6 class IIb. Accordingly, BUF reduced the signal strength of Sub^Ac^ by approximately half as well since it practically did not inhibit the deacetylase activity of HDAC class I. Finally, neither UF010 nor TAC and BUF affected the signal level in the case of Sub^Tfa^.

The selectivity of the inhibitors TMP269 and Cmpd13 for HDAC class IIa was manifested in an almost complete drop in the fluorescent signal upon incubation with Sub^Tfa^ and in the inalterability of fluorescent signal level in the case of Sub^Ac/Pro^ (Figure 2). Thus, it can be asserted that using the three class-selective substrates that we selected, namely Sub^Ac/Pro/Tfa^, the expected class selectivity of the five listed HDACi was unambiguously confirmed.

The next selective inhibitor of HDAC6/10 class IIb that we tested, Tubastatin A (TUBA) [38], unexpectedly caused suppression of the fluorescent signal of all three substrates to approximately the same extent (Figure 2). This discouraging result was of a fundamental nature and required further study and explanation.

#### 3.2.3. Validation of s^3^CTS Using Non-Selective HDACi

**Figure 3:**
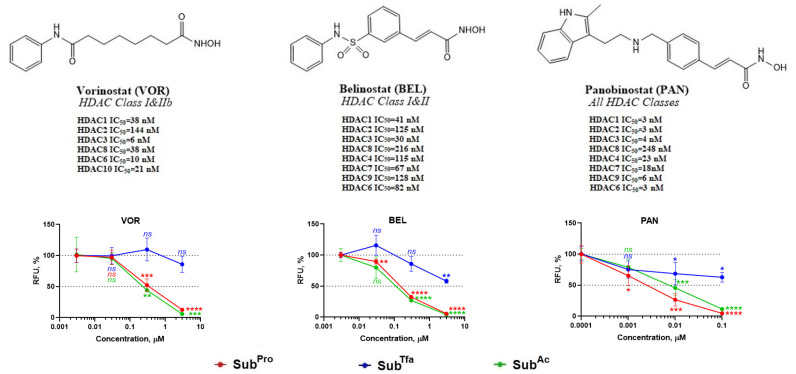
Structures with published IC_50_ data and results of testing non-selective HDACi with s^3^CTS. Statistical significance assessed by ANOVA with Dunnett’s post hoc test: **** (*p* < 0.001), *** (0.001 < *p* < 0.01), ** (0.01 < *p* < 0.05), * (0.05 < *p* < 0.1), and ns—not significant.

As part of further validation of the method, non-selective and pan-selective HDACi were tested—Vorinostat (VOR), Belinostat (BEL), and Panobinostat (PAN) (Figure 3). We observed a good agreement between the results of in-cell testing and the data obtained on recombinant enzymes [39], which was most clearly demonstrated in the case of VOR. This inhibitor that was inactive against HDAC class IIA in vitro also did not suppress Sub^Tfa^ deacylation in cell (Figure 3). Since VOR, like TUBA, is a strong inhibitor of HDAC6/10, it became clear that the deviations in the performance of the test system observed in the case of TUBA are related to its structural features and not to HDAC selectivity.

In addition to selectivity, s^3^CTS made it possible to correctly compare the potencies of different HDACi with each other. In accordance with the results obtained, the most active among the three HDACi studied was PAN, which is in a good agreement with the literature data (Figure 3). Occasionally, there were discrepancies for some compounds in the relative strength of inhibition of HDACs of different classes with the literature values, which could be due to differences between conditions of s^3^CTS and in vitro testing for HDACi.

### 3.3. Studying the Possible Causes of Errors in s^3^CTS Performance

#### 3.3.1. The Combination of TUBA and Sub^Acyl^ Does Not Cause Inhibition of HDACs Class I

**Figure 4:**
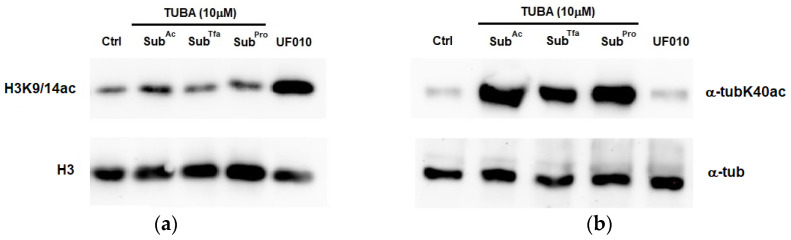
Western blot analysis of the inhibitory effect of TUBA on HDACs in the presence of Sub^Ac/Pro/Tfa^. (**a**) Accumulation of histone H3K9/14ac, the substrate of HDACs class I. (**b**) Accumulation of α-tubulineK40ac, substrate of HDAC6 class IIb; HCT116 cells were incubated for 24 h in the presence of TUBA (10 µM) and Sub^Acyl^ substrate (30 µM). HDAC class I inhibitor UF010 (10 µM) was taken as a positive (**a**) or negative (**b**) control.

As a possible reason for the incorrect performance of s^3^CTS, we initially tested a version with direct suppression of HDAC class I activity using a combination of TUBA and Sub^Pro^. The results of Western blot analysis showed that the simultaneous presence of TUBA with any of the three Sub^Acyl^ substrates in the cellular medium for 24 h did not lead to the accumulation of the substrate protein, namely the acetylated form of histone H3K9/14ac [28]; that is, the activity of HDAC class I was not inhibited in any of these cases (Figure 4a). A similar experiment performed with HDAC6 as a positive control confirmed the normal accumulation of the corresponding substrate protein, namely the acetylated form of α-tubulineK40ac in the presence of TUBA [28] and each of Sub^Acyl^ substrates (Figure 4b). Thus, the deviation from the expected selectivity of TUBA in the presence of Sub^Pro^ cannot be explained by HDAC class I inhibition.

#### 3.3.2. MBLAC2 Activity Affects Performance of s^3^CTS

A zinc-dependent enzyme, metallo-β-lactamase domain-containing protein 2 (MBLAC2), possessing palmitoyl-CoA hydrolase activity [40], was recently identified as an off-target for several hydroxamic HDACi, including TUBA [41]. We decided to investigate whether there is a connection between the incorrect performance of s**^3^**CTS and extra activity of HDACi against MBLAC2.

**Figure 5:**
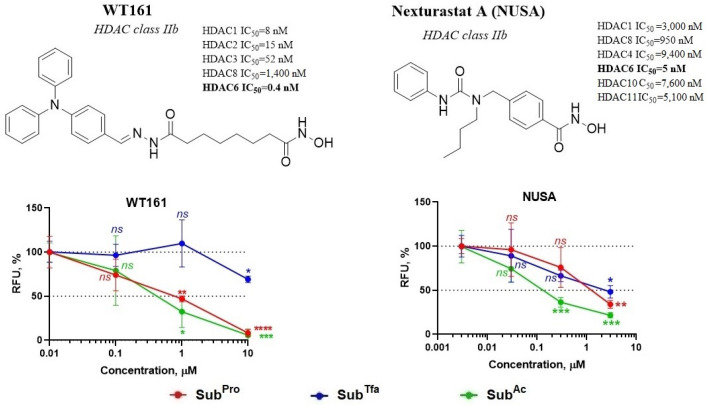
Structures with published IC_50_ data and results of selective HDAC6i testing with s^3^CTS. Statistical significance assessed by ANOVA with Dunnett’s post hoc test: **** (*p* < 0.001), *** (0.001 < *p* < 0.01), ** (0.01 < *p* < 0.05), * (0.05 < *p* < 0.1), and ns—not significant.

To increase the sample of tested compounds known to have activity against MBLAC2, according to [41], we chose two selective HDAC6 inhibitors for further work—WT161 [42] and Nexturastat A (NUSA) [43]. Testing showed that the selectivity of the inhibitory effect against HDACs for both compounds was determined incorrectly—in the case of Sub^Ac^ and Sub^Pro^, a sharp drop in the fluorescent signal was observed, which is more typical for pluripotent HDACi (Figure 3); in addition, in the case of NUSA, a twofold decrease in the Sub^Tfa^ signal was registered (Figure 5).

#### 3.3.3. Correlation between s^3^CTS Performance and MBLAC2 Inhibition

Hypothetically, the cause of deviations from the normal performance of s**^3^**CTS could be the influence of TUBA/WT161/NUSA (i) on the active transmembrane transport of Sub^Acyl^ and/or Prod^Lys^ [44], (ii) on the enzymatic modification of Prod^Lys^, such as N^ε^-methylation [33], or (iii) on the storage of Prod^Lys^ in lysosomes, etc. On the other hand, all these processes must be a consequence of inhibition of an off-target, most likely some metal-dependent enzyme. Obviously, the positive identification of MBLAC2 as a pivotal player would greatly facilitate the search for cellular processes responsible for s^3^CTS malfunctions.

To assess how reliably the performance of s**^3^**CTS is related to the inhibition of MBLAC2, we calculated the Spearman’s rank correlation coefficient for a small sample of HDACi that we had available (*n* = 8; Table 1). The initial ranking of compounds was carried out according to the affinity of the interaction between HDACi and MBLAC2 (pK_d_^app^) obtained by the chemoproteomics competition assay [41]. Among the eight HDACi for which pK_d_^app^ values were known, one was an aminoanilide (TAC), and seven were hydroxamic derivatives of benzoic acid (TUBA and NUSA), phenylacetic acid (BUF), cinnamic acid (BEL and PAN), and suberic dicarboxylic acid (VOR and WT161). The second ranking of HDACi was carried out according to the parameter of correctness of testing results, using a two-digit scale of “0” and “1”—correct and incorrect, respectively (Table 1).

The calculated Spearmen rank correlation coefficient was found to be *r_s_* = 0.901, which for the presented sample (*n* = 8) corresponded to the Student’s reliability criterion *t_r_* = 5.076 and meant the presence of a strong correlation (*p* < 0.01). Thus, we attained a formalized mathematical argument that MBLAC2 activity is essential for proper HDACi testing, and vice versa, a deviation from the known selectivity of HDAC inhibition under s^3^CTS conditions very probably is associated with suppression of MBLAC2 activity.

It was shown that pharmacological inhibition of MBLAC2 induced the biogenesis of exosomes and led to their accumulation in the cell culture supernatants [41]. On the other hand, biogenesis of exosomes is an initial step in exosome-mediated drug efflux that protects cancer cells from the toxic effects of drugs [45,46]. Considering the extra activity of TUBA/WT161/NUSA as inhibitors of MBLAC2, it cannot be excluded that these compounds may intensify the exosome-mediated efflux of hydrophobic Sub^Acyl^ substrates from the cell, thereby reducing their accessibility to histone deacetylases. In this case, the drop in the fluorescent signal for all three substrates observed by us (Figure 2 and Figure 5) would find its explanation. This issue is currently under study.

## 4. Conclusions

In this article, we confirmed the previously known substrate selectivity of Boc-Lys(Pro)-AMC for recombinant class I HDACs under in cell testing conditions for class-selective HDACi. Based on these results, we developed an improved testing protocol using three substrates, namely Boc-Lys(Pro/Tfa/Ac)-AMC, which provided a clear conclusion about the selectivity of a particular HDACi. However, if some HDACi had additional inhibitory activity against the zinc-dependent palmitoyl-CoA hydrolase MBLAC2, then the test system was likely to fail. We believe that this problem can be solved by creating a specialized cell line in which the main acyl-CoA thioester hydrolase activity is carried out by the ACOT family of hydrolases, which utilizes the Ser-His-Asp catalytic triad [47].

## Figures and Tables

**Table 1:** Ranking of tested HDACi.

HDACi	pK_d_^app^ *	Rank 1	Incorrect	Rank 2
BUF	<4.52	2.5	0	3
TAC	<4.52	2.5	0	3
VOR	<4.52	2.5	0	3
BEL	<4.52	2.5	0	3
PAN	5.86	5	0	3
WT161	6.04	6	1	7
TUBA	6.31	7	1	7
NUSA	7.59	8	1	7

* Data from [41]. BUF, Bufexamac; TAC, Tacedinaline; VOR, Vorinostat; BEL, Belinostat; PAN, Panobinostat; TUBA, Tubastatin A; NUSA, Nexturastat A; pK_d_^app^ = −lg K_d_^app^ (apparent dissociation constant); Incorrect, the incorrect performance of s**^3^**CTS.

## Data Availability

The data presented in this study are available in article.
